# Editorial: Artificial intelligence in bioimaging and signal processing

**DOI:** 10.3389/fphys.2023.1267632

**Published:** 2023-08-11

**Authors:** Seongyong Park, Abdul Wahab, Muhammad Usman, Imran Naseem, Shujaat Khan

**Affiliations:** ^1^ Cancer Data Science Laboratory, Center for Cancer Research, National Cancer Institute, National Institutes of Health (NIH), Bethesda, MD, United States; ^2^ Department of Mathematics, Nazarbayev University, Astana, Kazakhstan; ^3^ Department of Computer Engineering, College of IT Convergence Engineering, Chosun University, Gwangju, Republic of Korea; ^4^ School of Electrical, Electronic and Computer Engineering, The University of Western Australia, Crawley, WA, Australia; ^5^ College of Engineering, Karachi Institute of Economics and Technology, Karachi, Pakistan; ^6^ R&D Alexa Translations, Toronto, ON, Canada; ^7^ Love for Data, Karachi, Pakistan; ^8^ Siemens Healthineers, Princeton, NJ, United States

**Keywords:** artificial intelligence, biomedical imaging, bio-signal processing, AI applications, monitoring physiological events

## Introduction

This Research Topic explores the advancements in artificial intelligence (AI) in the realm of bioimaging and bio-signal processing. It encompasses a diverse range of studies spanning various bio-signal modalities and subspecialties. These modalities aim to monitor physiological events in patients, enabling applications such as fetal distress diagnosis, bone mineral density prediction, portable ECG measurements, pulse wave velocity estimation, drowsiness detection, knowledge extraction, and semantic understanding of bio-signals. The studies included in this compilation were specifically chosen for their focus on AI applications that have the potential to revolutionize these respective fields.

## Fetal distress diagnosis (FDD) using cardiotocography (CTG)

Zhang et al. introduced a novel multi-modal information fusion (MMIF) framework aimed at enhancing fetal wellbeing evaluation in late pregnancy and preventing sudden fetal death. This study encompasses several innovative contributions. Firstly, the authors employed the Category Constrained-Parallel ViT (CCPViT) approach to model unimodal representations for Gramian Angular Field (GAF)-based 2D images and label texts. This allowed for effective representation learning within each modality. Secondly, to address the challenge of misalignment between the two modalities, the authors proposed the Multimodal Representation Alignment Network (MRAN). MRAN employs a combination of constraint term loss and captioning loss. The constraint term loss ensures alignment between the image and text modalities, while the captioning loss promotes the generation of accurate text descriptions for a given representation. The final loss function is a convex combination of these two loss components. The image encoder component of the trained multimodal alignment network is subsequently utilized for the classification of GAF-based 2D images in a test dataset. The results demonstrate that the image encoder, trained with multimodal input data, surpasses state-of-the-art performance in the task of fetal distress diagnosis. While the model’s explainability in a broader sense is limited due to the scarcity of available labeled data for GAF 2D images in practical settings, the approach itself exhibits high potential to revolutionize the field of fetal distress diagnosis, making it more reliable and explainable.

## Bone mineral density (BMD) prediction in computational tomography (CT)

Kang et al. proposed a deep learning framework for estimating bone mineral density (BMD) from an axial cut of the L1 bone on CT images. This approach has the potential to eliminate the need for additional radiographic exposure in patients who have already undergone CT imaging but require quantitative measurements using dual-energy X-ray absorptiometry (DXA) or dual-energy CT. The proposed approach offers several advantages, including the ability to provide explainability by referencing anatomical context through CT images, automatic or routine screening using an established database, and cost savings. In this study, the authors first employed a residual U-Net to segment the total L1 bone or L1 vertebral body from the CT image. Subsequently, a residual CNN model was trained by feeding the original image, entire-vertebrae-masked images, or vertebral-body-masked images to predict the BMD measured by DXA for the same patient. To explain the BMD estimation results of the proposed model, the authors developed two explainable AI techniques called “Grad-RAM.” and “Grad-RAMP”. The performance of the model in predicting BMD demonstrated a good correlation with reference DXA-based measurements (0.85–0.90). The activation visualization using Grad-RAMP correctly captured the bone region and provided a clear visualization of bone density for a given CT image. Although this study was conducted in a single medical institution, its significance in clinical practice lies in its potential to not only provide cost savings and reduce radiologic exposure for patients but also enhance understanding of bone health within a given anatomical context.

## Arterial stiffness estimation using photoplethysmography (PPG) or blood pressure (BP)

Vargas et al. suggested machine learning methods for monitoring carotid-to-femoral pulse wave velocity (CF-PWV) to estimate arterial stiffness. They used a time-frequency spectrogram representation produced from photoplethysmography (PPG) or blood pressure (BP) acquired from a single non-invasive peripheral pulse wave signal instead of one-dimensional signals. The semi-classical signal analysis approach, Law’s mask for texture energy extraction, and the central statistical moments were used to extract features for the machine learning models. A reliable estimate of CF-PWV is critical for assessing arterial stiffness since it is the most important risk marker for identifying various cardiovascular diseases. Despite its widespread use in clinical practice, CF-PWV assessment is regarded as a difficult and error-prone activity for clinicians. Machine learning algorithms have shown considerable potential in this regard. Although *in silico* data was employed in this work, the results show the potential of an automated AI-based, efficient assessment of arterial stiffness utilizing *in vivo* data.

## Evolution of the artificial intelligence studies in electrocardiogram (ECG)

Yang et al. presented a comprehensive overview of the state of the art in AI-assisted electrocardiogram (ECG) research. The most recent papers on AI-assisted ECG research have been reviewed from a bibliographic standpoint to reveal trends in this field. A co-citation reference cluster knowledge visualization domain map was used to investigate the evolution of research hotspots. The Web of Science Core Collection (WoSCC) was used as the search data source. There were 2,229 papers examined, with 2,124 being original articles and 105 being review articles. The study did not include any clinical scenarios. AI-assisted ECG analysis has been used to diagnose a variety of cardiac and cardiovascular disorders, including myocardial ischemia and arrhythmias, and has shown promise when paired with other computational approaches. Further research on AI-assisted ECG analysis methods in various types of disorders, application settings, and cohorts is needed.

## FPGA based solution for portable ECG application

Liu et al. presents a fully-mapped FPGA accelerator for analyzing electrocardiogram (ECG) data using artificial intelligence (AI). The accelerator includes a fully-mapped 1-D convolutional neural network (CNN) and a fully-mapped heart rate estimator, serving as a dual-function analysis system. Each layer of the 1-D CNN is mapped to a specific hardware module on an Intel Cyclone V FPGA, and a virtual flatten layer is introduced to connect the feature extraction and fully-connected layers effectively. The design maximizes computational parallelism to accelerate CNN inference. The heart rate estimator performs pipelined transformations, self-adaptive threshold calculation, and heartbeat count on the FPGA without multiplexed resource usage. The heart rate calculation is optimized to eliminate division and achieve hardware-friendly implementation. Experimental results demonstrate that the accelerator achieves significant speedup compared to software implementations on ARM-Cortex A53 quad-core processor and Intel Core i7-8700 CPU. Notably, the accelerator exhibits high energy efficiency, surpassing existing studies, making it suitable for resource-limited applications such as wearable and portable ECG monitoring devices.

## Searching for semantic understanding in bio-signal

The development of compact and energy-efficient wearable sensors has made bio -signals more accessible. Analyzing these continuously recorded and multidimensional time series is important, and unsupervised data segmentation is a promising approach. Traditional change-point detection algorithms have limitations, such as requiring the complete time series and struggling with multidimensional data. To overcome these challenges, Strommen et al. proposed a new algorithm called Latent Space Unsupervised Semantic Segmentation (LS-USS). The LS-USS uses an autoencoder to learn a 1-dimensional latent space for change-point detection in multidimensional time series. To enable real-time segmentation, two algorithms are introduced: Local Threshold Extraction Algorithm (LTEA) and “batch collapse.” LTEA detects change-points when the computed metric exceeds a threshold, while “batch collapse” divides streaming data into manageable batches. By combining these algorithms, the proposed approach accurately segments time series data in real-time, making it suitable for applications that require timely change detection. Evaluations show that the LS-USS outperforms other state-of-the-art change-point detection algorithms in both offline and real-time scenarios when applied to various real-world datasets.

## Driving drowsiness detection using electroencephalography (EEG)

Drowsy driving is a major cause of road accidents worldwide. Detecting drowsiness early and accurately can greatly improve road safety. In an study Arif et al. presented a passive brain-computer interface (pBCI) system that uses electroencephalography (EEG) signals to accurately detect drowsiness during driving tasks. EEG data from 12 subjects were collected from specific regions of the brain, and spectral signatures of different brain rhythms were extracted. Various machine learning classifiers were employed to classify drowsiness based on the extracted features. The optimized ensemble model achieved the best classification results, and the F8 electrode position in the right frontal cortex was found to be the most effective for drowsiness detection. The proposed pBCI system offers several advantages, including reduced feature extraction cost, computational complexity, and physical intrusiveness. It has the potential for practical applications that require accurate and non-disruptive drowsiness detection using EEG signals.

## Conclusion

In conclusion, while artificial intelligence has witnessed remarkable advancements in various fields, its application in biomedical imaging and bio-signal processing calls for further focused efforts. The current Research Topic represent valuable steps towards realizing AI’s potential in these critical areas of healthcare. By staying at the forefront of these developments, we can expect significant improvements in medical diagnostics, treatment, and overall public wellbeing. Embracing the power of AI in biomedical and bio-signal processing will undoubtedly pave the way for a brighter and healthier future for humanity.

